# The Contingent Payoff of Transparency: The Mediating Roles of Citizen Participation and Enforcement in Health Service Delivery Improvement

**DOI:** 10.1111/jep.70401

**Published:** 2026-03-09

**Authors:** Samuel Atiku, Alfred Okoh, Olufisayo Olakotan

**Affiliations:** ^1^ Research Services Aston University Birmingham Birmingham UK; ^2^ Digital Technology and Innovation University of Staffordshire Stoke‐on‐Trent UK; ^3^ Department of Management, School of Business Management Baze University Abuja Nigeria; ^4^ Department of Neonatology, Women and Children's Directorate University Hospitals Leicester NHS Trust Leicester UK

## Abstract

**Background:**

How transparency reforms affect frontline health service delivery remains uncertain. Research presents a transparency paradox, where some studies report service improvements when transparency is combined with oversight, while others find minimal impact on outcomes such as staff absence or medicine availability.

**Objective:**

This article addresses the conditions under which transparency improves health services. We argue that inconsistent findings stem from construct slippage, where distinct reforms are grouped together. We propose an unbundled conceptualisation distinguishing three types: Passive Transparency (information only disclosure), VOICE Interventions (disclosure plus citizen participation), and TEETH Interventions (disclosure linked to credible sanctions).

**Methods:**

We conducted a scoping review of studies from 2000 to 2025 following PRISMA guidelines. Database searches yielded 6636 records, resulting in 49 included experimental, quasi experimental, and observational studies. Each intervention was coded as Passive Transparency, VOICE or TEETH based on its actual components rather than author labels.

**Results:**

The evidence shows that Passive Transparency alone rarely shifts health service outcomes, though it can modestly increase citizen knowledge. Interventions that combine disclosure with empowered VOICE mechanisms show more consistent gains, such as large mortality reductions found in Uganda and Malawi. Similarly, TEETH interventions with credible enforcement, such as centralised drug procurement, produced large price reductions. Where enforcement was weak, transparency had little effect.

**Conclusion:**

The impact of transparency on health service delivery is conditional rather than automatic. The transparency paradox is largely resolved when interventions are unbundled. Transparency functions as a diagnostic input whose effectiveness depends on being coupled with functional accountability arrangements: either empowered citizen VOICE or credible state TEETH.

## 1 Introduction

How transparency reforms alter everyday conditions inside clinics remains uncertain. Existing research reports gains in service delivery when transparency is combined with forms of oversight or citizen participation [1, 2, 3, 4]. Other studies describe very different outcomes, in which medicines remain unavailable, queues remain long, and staff absence continues despite new information rules [5, 6]. This contrast leads to the central question of this article: what are the conditions under which transparency interventions contribute to concrete improvements in health services and outcomes?

We argue that part of this inconsistency reflects a conceptual problem that we refer to as construct slippage. Reviews of social accountability and fiscal openness describe a wide array of initiatives related to public information on resources and services [2, 7, 8]. Yet many such studies group these initiatives under a single label of fiscal transparency even when they differ sharply in institutional design, channels for contestation, and forms of enforcement. When disclosure of budget documents, community scorecards and sanction backed audits are treated as variants of the same reform family, the mechanisms through which they influence public officials and citizens become obscured. It then becomes difficult to identify why some interventions appear to shift clinical outcomes while others remain largely symbolic.

To address these gaps in the scholarship, we advance an unbundled conceptualisation that links transparency more explicitly to accountability arrangements. Building on Fox [8], we suggest that effects depend on whether disclosure is combined with structured channels for citizen VOICE or with credible administrative TEETH, recognising that both elements may coexist. We distinguish three categories. Passive Transparency refers to disclosure of health information such as budgets, prices or scorecards in relatively static formats, including printed reports, PDF files or web portals, where structured follow‐up is limited [4, 9, 10]. VOICE Interventions connect information with formal opportunities for patient and community participation, such as health committees, community scorecards and participatory budgeting [11, 12, 13, 14]. TEETH Interventions link disclosure to credible threats of administrative, legal or market sanction, including randomised audits, performance linked budget rules or procurement reforms [2, 15].

We use this framework to revisit what we term the transparency paradox in health. The article synthesises experimental, quasi experimental and carefully designed observational studies between 2000 and 2025 that connect fiscal flows and resource management to frontline clinical delivery. Viewed through this unbundled lens, the evidence suggests that Passive Transparency alone rarely shifts health outcomes, although modest effects on intermediate indicators sometimes appear. Interventions that join disclosure with empowered VOICE or credible TEETH show more consistent, yet still context dependent, gains in service quality and health results. The available studies remain uneven in coverage, so any generalisation must be cautious. Even so, we propose that fiscal transparency is better understood as a diagnostic input within accountability systems, whose consequences depend on the strength and configuration of participatory and enforcement arrangements surrounding clinics.

This review builds upon and extends existing syntheses of social accountability and fiscal openness. While previous reviews have mapped the broad landscape of transparency, our contribution is distinct in two primary ways. First, we apply a more rigorous set of inclusion criteria that requires not only a specific fiscal information component, such as budgets, procurement data or audit findings, but also a measurable health service or clinical outcome. This approach deliberately excludes studies limited to soft outcomes like perceptions of trust or corruption, which often dominate social accountability literature but do not always correlate with tangible clinical improvements. Second, we address the problem of construct slippage by systematically coding interventions based on their actual design components rather than author assigned labels. Unlike existing health sector reviews that may treat transparency as a uniform intervention, our unbundled approach distinguishes between Passive disclosure, VOICE based participation, and TEETH backed enforcement. This granular coding allows us to resolve the transparency paradox by identifying the specific institutional configurations that allow information to translate into service gains. Due to the extreme heterogeneity of intervention types, ranging from community based maternal health trials to national pharmaceutical procurement reforms, a formal meta analysis was not feasible. Instead, we provide an interpretive synthesis that maps the conditional payoff of these reforms across diverse global contexts.

## 2 Unbundling Accountability and a Conceptual Framework for Health Systems

The central transparency puzzle introduced earlier, in which a high quality trial in Indonesia reports null effects [5] while a comparable intervention in Malawi shows marked improvements [16], cannot be fully resolved if transparency is treated as a single intervention. When informational inputs and accountability mechanisms are collapsed into one category, variation in outcomes appears erratic and the institutional pathways that connect fiscal disclosure to clinical change remain obscure. We therefore propose an unbundled conceptualisation that distinguishes three non interchangeable intervention types.

The first is Passive Transparency, which we define as an information only model, sometimes described as a transparency placebo. Here, reform is limited to the disclosure of data such as clinic budgets, audit findings or drug price lists, often in static formats such as web portals or notice boards, with limited structured follow up [4, 10]. The associated theory of change usually relies on a selection pathway, in which patients use the data to choose better providers, or a reputational pathway, in which providers correct behaviour to avoid embarrassment. Our reading of the empirical record suggests that both pathways frequently fail, because many patients are unaware of the information or do not consider it salient, and providers often face no material consequence if they ignore reputational pressure.

A second type is a VOICE mechanism, where disclosure is intentionally combined with structured spaces for citizen participation, including community scorecards, health committees and participatory budgeting forums [11, 12, 14, 16]. In these models, information functions as a shared reference point that can structure disagreement and negotiation, while the participatory forum creates at least some expectation of follow through. Evidence suggests that impact is more likely when these forums have clear mandates, regular meetings and links to decision making authority, although such conditions are not always present.

The third intervention type is a TEETH mechanism, which we characterise as state‐led accountability. Here, transparency is bundled with a credible prospect of formal sanction. The central logic is not deliberation from below but deterrence from above. Public audits that trigger investigations or affect electoral prospects, watchdog monitoring that carries a real threat of exposure and procurement rules such as volume based purchasing arrangements that make continued access to public contracts conditional on compliance all fall into this category [4, 15]. In these models, information about budgets, contracts or performance is explicitly linked to administrative, legal or market consequences. Providers and officials may adjust behaviour not because they have agreed to change in dialogue with citizens, but because the expected cost of non‐compliance has increased. The available evidence suggests that such mechanisms can reduce corruption and improve selected health outcomes, although their operation is sensitive to political and bureaucratic constraints.

These intervention types can be expressed in simple form as

Impact=T×max(Mvoice,Mteeth)×C.

This expression is a **heuristic** representation of the causal logic in the reviewed studies, not an estimated statistical model. We use max (Mvoice, Mteeth) to reflect that most interventions have one dominant accountability pathway—either participatory pressure (VOICE) or sanction‐backed deterrence (TEETH). In practical terms, strong disclosure (*T*) with negligible VOICE and TEETH will usually produce little observable service improvement.

Where Impact denotes measurable change in a health service outcome, *T* represents the transparency input, Mvoice and Mteeth represent the strength of the patient‐led or state led accountability mechanism, and C captures the contextual and capacity conditions that shape implementation. In this formulation, Passive Transparency corresponds to cases where Mvoice = 0 and Mteeth = 0, so that even substantial increases in T yield no effect on Impact.

Studies classified as failed VOICE interventions are those in which participatory spaces exist but lack authority, are not properly resourced or are disconnected from information, which implies that the effective value of *M* is very close to zero. TEETH mechanisms without reliable enforcement, for instance when audits are ignored or sanctions are rarely applied, similarly generate low values of *M*.

The framework implies that marked and durable impact is only likely when the transparency input is multiplied by a functional accountability mechanism, whether VOICE or TEETH, and when contextual conditions do not neutralise that mechanism. It also suggests that the literature is less inconsistent than it first appears. Passive interventions cluster near zero on our impact metric, while studies that combine disclosure with empowered VOICE or credible TEETH tend to show patterned, though context dependent, improvements. In the remainder of the article, we use this framework as an analytical lens. We examine Passive Transparency, VOICE and TEETH interventions in turn, and in Section Results, 4 we bring together experimental, quasi experimental and observational evidence to assess how far this unbundled approach can account for the mixed results reported in earlier work.

### 2.1 Illustrating the Unbundled Framework

To clarify the practical application of our conceptual framework, we provide a concrete example for each category within the health system context. Passive Transparency is illustrated by a government web portal that publishes clinic budgets or drug price lists without any formal mechanism for follow up or public engagement. A VOICE intervention is exemplified by a community scorecard programme where patients meet regularly with clinic staff to discuss performance data and agree on specific service improvements. Finally, a TEETH intervention is represented by a centralised procurement system where transparent tender data are used to identify and automatically sanction suppliers who fail to meet price or quality benchmarks.

In plain language, the heuristic formula suggests that even high quality information will fail to improve health services if it is not coupled with either a functional channel for citizen participation or a credible threat of state enforcement. Specifically, if both the voice and teeth mechanisms are negligible, the final impact on clinical outcomes will remain near zero regardless of how much transparency is provided. This approach allows practitioners to move beyond simply asking what data should be published and instead focus on who can act on that information and with what authority.

## 3 Methodology

Our approach treats the transparency literature as a structured dataset for analysing how fiscal information influences health service delivery. The review operationalises the conceptual model in Section Objective, 2 by coding whether interventions contain Passive transparency (information disclosure only), and whether they also include VOICE mechanisms (structured participation and collective problem‐solving) and/or TEETH mechanisms (enforceable rules, sanctions or binding compliance requirements). This scoping review follows established guidance [17, 18], which is well suited to mapping a heterogeneous evidence base while applying systematic coding of intervention components and pathways. Reporting adheres to core elements of PRISMA [19].

We conducted structured searches in three major databases, Scopus, PubMed and Web of Science, for peer reviewed studies published between 1 January 2000 and 30 October 2025. Details of the database search strategy, along with the search results, are provided in Table 1.

**Table 1:** Database search strategy and results.

Database	Search string	Number of record
SCOPUS	(“fiscal transparency” OR “budget transparency” OR “budget disclosure” OR “open contracting” OR “public procurement” OR “pharmaceutical procurement” OR “drug price*“ OR “medicine price*” OR “drug procurement” OR “public audit*” OR “audit report*” OR “citizen report card*” OR “community score card*” OR “social accountability” OR “volume based procurement” OR “volume based purchasing” OR “centralized procurement” OR “centralised procurement”) AND (health OR clinic* OR hospital* OR “health service*” OR “health system*” OR “maternal health” OR “newborn health” OR “child health” OR “reproductive health” OR “primary health care” OR “primary care”) AND NOT (corporate OR “stock market” OR “shareholder*” OR “listed company” OR “financial reporting” OR “bank disclosure”)	4483
Web of science	https://www.webofscience.com/wos/woscc/summary/50b81263-7337-4ee4-a28d-3ba5d2f23894-01893d60eb/relevance/1 (“fiscal transparency” OR “budget transparency” OR “budget disclosure” OR “open contracting” OR “public procurement” OR “pharmaceutical procurement” OR “drug price*“ OR “medicine price*” OR “drug procurement” OR “public audit*” OR “audit report*” OR “citizen report card*” OR “community score card*” OR “social accountability” OR “volume based procurement” OR “volume based purchasing” OR “centralized procurement” OR “centralised procurement”) AND (health OR clinic* OR hospital* OR “health service*” OR “health system*” OR “maternal health” OR “newborn health” OR “child health” OR “reproductive health” OR “primary health care” OR “primary care”) NOT (corporate OR “stock market” OR “shareholder*” OR “listed company” OR “financial reporting” OR “bank disclosure”)	354
PubMed	“(“fiscal transparency”[tiab] OR “budget transparency”[tiab] OR “budget disclosure”[tiab] OR “open government”[tiab] OR “open contracting”[tiab] OR “social accountability”[tiab] OR “public accountability”[tiab] OR “public reporting”[tiab] OR “citizen report card*”[tiab] OR “community score card*”[tiab] OR (“public procurement”[tiab] AND (transparency[tiab] OR accountability[tiab])) OR (“pharmaceutical procurement”[tiab] AND (transparency[tiab] OR accountability[tiab])) OR (“drug procurement”[tiab] AND (transparency[tiab] OR accountability[tiab]))) AND (“health services”[MeSH] OR “maternal health services”[MeSH] OR “child health services”[MeSH] OR “primary health care”[MeSH] OR “pharmaceutical services”[MeSH] OR “health services”[tiab] OR “health service”[tiab] OR “health system*”[tiab] OR “health care”[tiab] OR clinic*[tiab] OR hospital*[tiab]) AND (“2000/01/01”[dp]: “2025/04/30”[dp]) NOT (corporate[tiab] OR “stock market”[tiab] OR shareholder*[tiab] OR “listed company”[tiab] OR “financial reporting”[tiab])”	1799
		6636

The starting point was chosen to capture the emergence of social accountability interventions in the early 2000s and later digital platforms. Search strings combined three blocks of terms. The first block covered fiscal transparency and accountability instruments, including budget disclosure, open contracting, public audits, citizen monitoring, scorecards and participatory budgeting. The second block focused on health service delivery, including clinics, hospitals, primary care, maternal and child health, pharmaceuticals, and broader public health systems. The third block contained exclusion terms that removed work on corporate disclosure and private sector reporting, and on purely political outcomes such as voting or attitudes without any service dimension. We also complemented database searches with citation tracking of core transparency reviews in public finance and health governance.

The initial search produced 6636 records. After removal of 1178 duplicates, 5458 unique items remained. Two reviewers independently screened titles and abstracts using pre agreed criteria, working in two rounds to minimise drift in judgement. Inter rater agreement, measured with Cohen's *κ*, was high, and disagreements were resolved through discussion. This stage identified 210 articles for full text assessment. Three reviewers then examined full texts against detailed inclusion and exclusion criteria that reflected our concern with construct precision. Studies were eligible when they evaluated a public sector intervention with an explicit fiscal information component and reported at least one observable outcome related to health service delivery, health outcomes or health related fiscal performance, such as drug prices, procurement delays or facility level budget execution. Studies that focused only on perceptions of corruption or trust were retained only when they also reported concrete service indicators. Interventions centred on sanctions, audits or participation without any transparency component, and studies of corporate or private sector disclosure, were excluded. Table 2 summarises the inclusion and exclusion criteria. The final sample consists of 49 studies.

**Table 2:** Inclusion and exclusion criteria.

Dimension	Inclusion criteria	Exclusion criteria
Intervention	Public sector programmes or reforms that disclose fiscal information related to health or health relevant services, such as budgets, procurement data, audit findings or facility scorecards.	Corporate reporting, private sector transparency initiatives or interventions without any fiscal information component.
Transparency component	Intervention contains an explicit component that releases or circulates fiscal information to citizens, providers or public officials.	Accountability or anti corruption interventions based only on sanctions, complaints or civic participation, without disclosure or use of fiscal information.
Outcome domain	Study reports at least one observable outcome on service delivery, health outcomes or health related fiscal performance, such as prices, contract completion or delay.	Studies that report only perceptions, attitudes or indices of trust or corruption, without any observable service or fiscal outcome.
Study design	Experimental, quasi experimental or observational designs with clearly described methods and outcome measures.	Purely theoretical pieces, opinion essays and descriptive accounts without identifiable outcome measures.
Sector and level	National or subnational public sector interventions that affect health services directly, or other services when health relevant fiscal outcomes are reported.	Studies of political transparency that focus only on elections, party finance or legislative behaviour, with no link to service delivery or health relevant fiscal outcomes.
Time period and language	Empirical studies published between 2000 and April 2025 in English.	Studies outside this period or in other languages when a full text translation was not available.

We undertook a structured appraisal of methodological quality. Quantitative and mixed method studies, including randomised controlled trials, quasi experimental designs and panel models, were assessed using the Mixed Methods Appraisal Tool (MMAT, 2018 version). These ratings were not used to exclude studies, but to assess the strength of causal claims in Section Results, 4.

Our data extraction and coding strategy was aligned with the conceptual model. We developed an extraction template, piloted it on a subset of studies and then applied it to the full data set. Descriptive fields recorded author, year, country, level of government, sectoral focus, intervention description, study design and outcome measures. We then coded each intervention according to whether it contained only Passive Transparency, or whether it also incorporated channels for Voice or mechanisms supplying Teeth. Crucially, codes were assigned based on intervention components, not author labels, as similar terminology often masked design differences. We also coded the causal pathways proposed by each study (e.g., electoral incentives, bureaucratic responses, sanctions) and recorded contextual features like administrative capacity and political competition. This strategy produced a structured data set to analyze how combinations of transparency, Voice, Teeth and context shape health service outcomes.

## 4 Results

**Figure 1:**
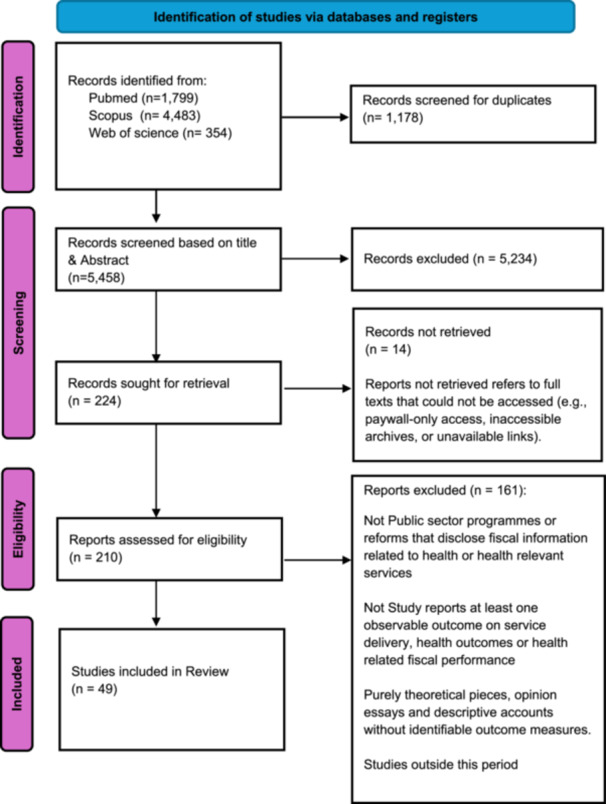
PRISMA‐ScR flow diagram.

Our systematic search identified 5458 unique records (after removing duplicates). After title, abstract and full‐text screening against predefined inclusion criteria, 49 empirical studies met the threshold for synthesis (see Figure 1 below).

### 4.1 Information Only Interventions and the Limits of Passive Transparency

Across the eight Passive studies, transparency is present in the form of measurable T, yet participation and sanctions, Mvoice and Mteeth, remain close to zero. In this configuration, primary health outcomes, service use, and procurement prices stay near baseline values, while gains appear mainly on intermediate measures such as knowledge and narrow prescribing changes. The two randomised trials on service delivery illustrate this pattern clearly. Arkedis and colleagues show that communities in Indonesia and Tanzania that receive audit findings on maternal and newborn health, plus supported planning but no extra authority or resources, record intention to treat effects that are small and statistically indistinguishable from zero [5]. Yu and colleagues report that a short leaflet given after COVID 19 vaccination raises correct knowledge by about 7.6 percentage points, while behaviour and clinical outcomes are not observed [20]. Knowledge changes and behaviour remains largely stable.

Evidence on procurement and prescribing behaviour points in the same direction. Kohler and Martinez [21] analyse Brazil's Banco de Preços em Saúde. They find that coefficients associated with use of the database are small and rarely reach conventional thresholds in statistical tests, which implies little systematic pressure on prices [22]. Cheng and colleagues compare local government purchases with the Philippine Drug Price Reference Index. Their Laspeyres index shows around four fifths of units buying above the benchmark and some products being purchased at more than twice the reference price [23]. Within facilities, the Hubei prescribing studies report that public posting of doctor level indicators on injections, antibiotics and costs produces a consistent reduction in injection use of about three to four percentage points [24, 25]. Effects on antibiotic use are absent or mixed across diagnoses, with some substitution away from one condition toward another [25, 26]. A mechanism study finds that only about 21.5% of surveyed patients are even aware of the bulletin boards. This awareness is not associated with higher perceived value or reported use once observable characteristics are taken into account [27]. See Table 3 for summary of the results.

**Table 3:** Passive transparency interventions, mechanism coding and quantitative findings.

References	Country/Setting	Sector/Population	Design	Transparency (T)	Mvoice	Mteeth	Primary outcome(s)	Quantitative findings
Arkedis et al. [5]	Indonesia, Tanzania	Maternal and newborn health	Cluster RCT	Dissemination of audit findings and health data	Low	0	Antenatal and postnatal service use	Estimates close to zero; no statistically reliable effect on service use.
Cheng et al. [23]	Philippines	Local government procurement	Observational	National Drug Price Reference Index	0	0	Drug procurement prices	80% of units purchased above benchmark; some prices twice the reference.
Kohler et al. [22]	Brazil	Public pharmaceutical procurement	Mixed effects models	Banco de Preços em Saúde online database	0	0	Unit purchase prices	Coefficients small and not statistically significant; no consistent price reductions.
Yu et al. [20]	China	Adult vaccine recipients	Individual RCT	COVID 19 health leaflet	0	0	COVID 19 knowledge	Knowledge increased by 7.6 percentage points; behavioural outcomes not measured.
Wang et al. [24]	China, Hubei	Hospital outpatient prescribing	Quasi experimental	Bulletin boards with doctor level indicators	0	0	Injection and antibiotic rates	Injections reduced by roughly 4%; no reliable change in antibiotic use or cost.
Zhang et al. [25]	China, Hubei	Hospital outpatient prescribing	Propensity matching	Same bulletin board intervention as Wang et al. [24]	0	0	Injection and antibiotic rates	Decrease in injections; estimates for antibiotics and cost close to zero.
Tang et al. [26]	China, Hubei	Hospital outpatients	Randomised trial	Same bulletin board intervention as Wang et al. [24]	0	0	Condition specific antibiotic rates	Antibiotics for gastritis reduced by 12.72 percentage points; hypertension use rose.
Chen et al. [27]	China, Hubei	Hospital patients	Matching and modelling	Exposure to bulletin boards with prescribing indicators	0	0	Information awareness and use	21.5% of patients aware; awareness not associated with higher reported use.

### 4.2 Voice Interventions and the Conditions Under Which Participation Amplifies Transparency

Across the 16 investigations that examine Voice, the evidence suggests a patterned interaction between disclosure of performance information and the institutional space through which citizens engage providers. The purpose of this section is to clarify how these elements jointly shape service outcomes. Transparency is consistently present, whereas participation, referred to as Mvoice, ranges from brief village discussions to more formalised assemblies. Authority over budgets, sanctions, or personnel, labelled Mteeth, is limited in most settings. This configuration generates wide variation in effects because the institutional channels that translate information into behavioural change differ substantially. Several studies record marked improvements in utilisation, service quality, or basic health indicators, although others produce precise null results even when participation is well organised. The following paragraphs examine these patterns and show how institutional arrangements condition the influence of Voice.

The clearest improvements emerge in intensive community monitoring settings in which disclosure and collective deliberation recur over time. In rural Uganda, communities and providers reviewed facility‐specific report cards through repeated discussion, and this combination of transparency and structured participation was associated with large reductions in mortality [11]. Under‐five deaths fell by roughly one third, infant mortality declined by a similar magnitude, and neonatal mortality dropped by about one half. These shifts coincided with higher utilisation and improvements in weight‐for‐age. The participation‐only arm, which lacked specific information, produced no measurable change. A related pattern is evident in Malawi, where the Community Score Card programme increased direct contact between households and providers. Home visits during pregnancy rose by about 20 percentage points and postnatal visits by roughly 6, and increases also appeared in contraceptive use, facility deliveries and reported satisfaction [16, 28]. At district level in Uganda, the CODES initiative produced substantial improvements in correct treatment for malaria, pneumonia and diarrhoea, with increases of 23, 19 and 13 percentage points respectively [12]. Taken together, these intensive interventions show that repeated discussion anchored in specific information can alter provider conduct across several domains. A limitation is that these findings arise from targeted regions and may not capture the diversity of administrative settings elsewhere.

A second group of moderate‐intensity scorecard initiatives yields measurable but more modest gains. In Ghana's insurance programme, community scorecards raised renewal rates and increased formal care‐seeking, although no changes appeared in satisfaction or waiting times [29]. Bangladesh's community clinic scorecards produced higher utilisation of antenatal, family planning and curative services, yet the magnitude of improvement remained limited [30]. Additional scorecard pilots in Ghana documented increases in drug availability and communication quality, with essential drug stocks rising from 62% to 84% although the absence of a counterfactual means the contribution of Voice cannot be isolated [29]. A similar pattern is visible in Uganda, where high‐intensity community scorecards raised several performance measures, such as increases in male ANC support and improvements in facility readiness scores, but, as [31] show, these gains were produced in a design lacking a comparison group and therefore cannot be attributed to strengthened Voice alone. These findings indicate that moderate‐intensity participation, when paired with performance information, can shape selected aspects of compliance behaviour and provider responsiveness. Broader changes in service coverage or health outcomes appear to require stronger institutional mechanisms that link citizen preferences to administrative action.

In contrast, a set of studies shows near‐zero effects despite the presence of participatory structures. In Uttar Pradesh, report cards combined with village and provider meetings produced minimal changes in antenatal care, delivery, or postnatal services, with estimates consistently close to zero [5]. In Burundi, strengthened facility committees displayed improvements in internal organisation but no shifts in utilisation, quality or managerial practices, aside from a rise in chief‐nurse turnover in the information arm [32]. Uganda's baraza meetings increased awareness of official responsibilities yet produced no statistically discernible changes in composite indicators for health or education [6]. These results indicate that participatory spaces without authority over resources or personnel provide limited incentives for providers to modify behaviour. It remains possible that longer time horizons or additional institutional support would generate different outcomes, and this marks a clear scope condition.

A final group of studies examines contexts in which Voice is linked to binding or semi‐binding authority over local spending. In Brazil, participatory budgeting provides citizens with a structured route to discuss spending priorities, and allocations are publicly disclosed. Municipalities adopting these procedures show reductions of five to ten per cent in infant and child mortality and modest increases in health and sanitation expenditure [33, 34]. These findings suggest that when communities possess some influence over fiscal decisions, their preferences can shape resource allocation in ways that support population‐level gains. The extent to which this evidence transfers to settings with weaker administrative capacity remains uncertain. Table 4 summarises the quantitative results across all 16 studies.

**Table 4:** Voice interventions, mechanism coding and quantitative findings.

References	Country/Setting	Sector/Population	Design	Transparency intervention (T)	Mvoice	Mteeth	Primary outcomes	Quantitative findings (Main effects)
Fabbri et al. [35]	Uttar Pradesh, India	Maternal and newborn health	2 × 2 cluster RCT; repeated cross‐sections	Report cards on MNH coverage shared in village and provider meetings	Low	0	ANC4+, facility delivery, PNC	Estimated effects on all primary indicators are small and statistically indistinguishable from zero.
Blake et al. [29]	Ghana	Maternal and newborn health	Before and after pilot	Community and facility scorecards on readiness and client experience	Medium	0 to low	Facility readiness; client experience	Facilities exhibit notable gains in readiness. Essential drug availability increases from 62% to 84% and client courtesy improves from 58% to 76%.
Opoku Duku et al. [36]	Ghana	Health insurance users	Cluster RCT	NHIS scorecards on staff behaviour, waiting times and claims	Medium	Low	NHIS renewal; care‐seeking	The programme increases NHIS renewal by 6.6 percentage points and formal care‐seeking by 7.3 percentage points.
Touchton and Wampler [33]	Brazil	Municipal population	Municipal FE panel	Participatory budgeting with public disclosure	High	Medium	Infant mortality; health spending	Municipalities adopting participatory budgeting show substantially lower infant mortality, with a reduction of roughly 23%.
Kiracho et al. [31]	Uganda (Kibuku District)	MNCH users	Quasi‐experimental	Community scorecards with joint action plans and interface meetings	High	Low	Facility delivery; ANC; PNC	Significant gains in men escorting wives for ANC and reductions in births delivered by traditional birth attendants. Facility readiness also strengthens.
Waiswa et al. [12]	Uganda; 16 districts	Child health (U5)	Cluster RCT	District and subcounty scorecards plus community dialogues	Medium	Low	Correct treatment of malaria, pneumonia and diarrhoea	Intervention districts achieve large improvements. Malaria treatment increases by 23 percentage points and pneumonia by 19 percentage points.
Hanifi et al. [30]	Bangladesh	Primary care	Quasi‐experimental with comparison clinics	Community clinic scorecards and revitalised committees	Medium	0 to low	ANC; PNC; FP; curative visits	Intervention clinics experience higher utilisation across several indicators including ANC and family planning compared to comparison clinics.
Gullo et al. [16]	Malawi	Reproductive health	Cluster RCT	Community score card cycles with community and provider engagement	High	Low	CHW visits; FP; facility delivery	The scorecard generates substantial increases in home visits and significantly higher modern contraceptive use and facility delivery.
Gullo et al. [28]	Malawi	Frontline health workers	Cluster RCT	CSC cycles focusing on teamwork and accountability	High	Low	Teamwork; accountability; readiness	The scorecard improves teamwork and perceived accountability among health workers and reduces stock‐outs for essential commodities.
Gonçalves [34]	Brazil	Municipal population	Municipal FE panel	Participatory budgeting	High	Medium	Infant and child mortality	Adoption is associated with 5%–10% lower infant and child mortality relative to non‐adopters.
Falisse and Ntakarutimana [32]	Burundi	Primary care	Cluster RCT	HFC training with PBF performance indicators	Medium	Low	Management; service quality; utilisation	Training leads to improved committee organisation but no measurable improvements in utilisation or perceived quality.
Mogues et al. [6]	Uganda	Health and education users	Cluster RCT	Public baraza meetings where officials present performance	High	0 to low	Composite service indicators	Meetings raise citizen awareness but show no statistically significant effects on health or education service delivery outcomes.
Björkman Nyqvist et al. [11]	Rural Uganda	Primary care; child health	Two cluster RCTs	Community and provider report cards; repeated interface meetings	High	Low	Under‐five, infant, and neonatal mortality	The information plus participation arm achieves large reductions in mortality. Under‐five deaths fell by 32% and neonatal deaths by 51%.

The magnitude of impact within the VOICE portfolio is most evident when comparing intensive and moderate interventions. High intensity programmes in Uganda achieved relative mortality reductions of approximately 32% for children under 5% and 51% for neonatal deaths. In Malawi, the community scorecard approach led to an absolute increase of 20 percentage points in home visits during pregnancy and a 6 percentage point rise in postnatal visits. Conversely, low intensity interventions in Uttar Pradesh recorded absolute changes of only 0.8 to 1.1 percentage points, which were statistically indistinguishable from zero.

### 4.3 Public Accountability and Sanction‐Backed Procurement Reforms (TEETH)

This reform cluster covers arrangements in which high transparency T is tied to enforcement sanctions with real financial consequences, such as exclusion of non compliant suppliers from public reimbursement. In our notation, T is high, Mteeth is clearly positive and Mvoice is close to zero. The performance of these interventions depends on how strong and credible enforcement actually is and on the incentives created by contextual conditions C around access and quality. We treat this configuration as a demanding setting for evaluating transparency in health systems, because disclosure is inserted into procurement and reimbursement rules that constrain behaviour rather than remaining a purely informational exercise.

The first set of cases, drawn from Latin America, India and China, shows what can happen when Teeth are applied firmly. Colombia's centralized purchasing of hepatitis C drugs, with guideline based case definitions and mandatory use of PAHO Strategic Fund regimens, reduced prices by more than 90 per cent and lowered total spending from about US 100 million to under US 10 million while preserving SVR12 cure rates around 95%–96% [37]. Brazil's federal purchase of rituximab for lymphoma, at a single national price for all SUS oncology hospitals, generated large estimated savings compared with fragmented hospital purchasing without clear evidence of reduced access [38]. In Delhi and Tamil Nadu, centralized tenders combined with essential drug lists cut the public drug bill by around 30% and raised medicine availability and EDL use [39, 40]. China's National Centralised Drug Procurement (NCDP) and National Volume‐Based Procurement (NVBP) programmes, which publish tender materials and tie hospital purchasing and reimbursement tightly to winning bids, form the most internally consistent expression of this model [41, 42, 43, 44, 45, 46, 47, 48, 49, 50, 51, 52, 53, 54, 55, 56, 57, 58, 59, 60, 61].

Across multiple drug classes and high cost devices, winning generic and device prices typically fall by 50%–90% and firm level analyses report higher profitability for successful bidders, which suggests that enforcement can align commercial incentives with purchasing rules in a durable way [42, 43, 44, 45, 46, 47, 48, 49, 50, 51, 52, 53, 55, 56, 57, 59, 60, 62]

A contrasting pattern appears where Teeth are weak, misdirected or inconsistently used, even when transparency is extensive. In Ethiopia and Tanzania, procurement rules that favour local manufacturers record and publish prices but place greater weight on industrial objectives than on patient affordability, and government purchasers at times pay more for local than imported products while patients face higher prices for local brands despite lower official mark ups [63]. Tanzania's NHIF reference price scheme, with public reimbursement ceilings and limited audit capacity, shows similarly weak pressure on pharmacies when ceilings sit above prevailing retail prices [64]. Evidence from China also reveals important limits. Price cuts for targeted items in NVBP and NCDP are often accompanied by higher volumes and spending on non targeted substitutes or by increased non drug charges at hospital level, so that the net effect on overall expenditure is uneven across facilities [41, 48, 49, 50, 61]. In studies of antibiotics, lower NVBP prices sometimes coincide with higher use, which raises concerns about overuse and quality of care that are not resolved by procurement reforms alone [54].

Viewed through the Impact = *T* × Mteeth × *C* framework, the pattern across these cases is internally consistent. Where transparency is embedded in credible, enforced procurement and reimbursement rules, as in Colombia, Brazil, Delhi, Tamil Nadu and the main NCDP and NVBP rounds in China, the literature reports large and sustained reductions in prices and public pharmaceutical spending, together with gains in availability or utilisation [37, 38, 39, 40, 41, 43, 44, 45, 46, 47, 48, 49, 50, 51, 52, 53, 55, 56, 57, 58, 59, 60, 61, 62].

Where Teeth are weak, oriented toward industrial goals or offset by local substitution and charging practices, transparency on its own leaves affordability and quality problems largely intact [48, 50, 54, 61, 63, 64].

In summary, transparency functions less as a self sufficient remedy than as a diagnostic input whose consequences depend on the strength, direction and execution of associated accountability mechanisms (see Table 5 for summary of findings). For TEETH interventions, results are primarily observed as relative price and expenditure reductions. Centralised drug procurement in Colombia achieved a relative price reduction of more than 90% for hepatitis C medications. Similarly, national volume based procurement programmes in China produced relative price cuts ranging from 50% to 90% across various drug classes. In terms of fiscal savings, pooled procurement in Delhi cut the public drug bill by a relative margin of approximately 30%. These standardised figures demonstrate that while Passive transparency yields negligible gains, interventions bundled with functional VOICE or TEETH mechanisms produce substantial shifts in clinical and fiscal performance.

**Table 5:** Public accountability (TEETH) interventions, mechanism coding and quantitative findings.

References	Country/Setting	Sector/Population	Design	Transparency intervention (T)	Mvoice	Mteeth	Primary outcome(s)	Main quantitative findings (Very brief)
Pérez et al. [37]	Colombia	HCV patients	Policy assessment	Centralised purchasing of DAAs through PAHO Strategic Fund	0	High	DAA prices; treatment cost	DAA prices fell by over 90%; total direct cost dropped from US100 million to US8.4 million.
Martins et al. [38]	Brazil	Lymphoma patients	Documentary analysis	Federal centralised purchasing of rituximab with published prices	0	High	Rituximab prices; expenditure	Central contracts secured 70% price discounts while sustaining patient access.
Chokshi et al. [40]	India	Public drug procurement	Comparative case study	State level pooled procurement with published tenders	0	High (TN)	Procurement prices; availability	Tamil Nadu obtained 100% of listed drugs; Bihar paid up to twice the price for common items.
Roy Chaudhury et al. [39]	India (Delhi)	Public hospitals	Retrospective evaluation	State drug policy with centralised pooled procurement and QA inspections	Low	High	Procurement costs; availability	Bulk EDL procurement cut drug costs by 30% and facility availability exceeded 80%.
Ewen et al. [63]	Ethiopia and Tanzania	Essential medicines	Adapt WHO/HAI survey	Procurement and retail price data distinguishing local vs. imported products	0	Low	Procurement and patient prices	Ethiopia paid more for local than imported products; Tanzania patients often paid more due to high mark ups.
Kirua et al. [64]	Tanzania	Private pharmacies	Cross‐sectional survey	Publication and use of NHIF reimbursement price list as reference	0	Low	Pharmacy retail prices	Reference prices exceeded pharmacy prices for half of medicines; others became more expensive.
Wang et al. [56]	China	Cancer patients	Interrupted time series	NVBP pilot for gefitinib with published winning prices	0	High	Purchase volumes; expenditure	Gefitinib volume doubled and spending fell by 40%; total drug volume rose by 57%.
Li et al. [53]	China	Cardiovascular care	Quasi‐experimental	Municipal volume price contracts recorded on provincial platform	0	High	DDD adjusted prices	Average adjusted prices fell by 41% in Xiangyang compared with the control city.
Chen et al. [50]	China	Hospital procurement	Interrupted time series	National pilot NVBP with publicly announced winning prices	0	High	Price and expenditure indices	NVBP produced large price reductions for bid winning generics and declines in total spending.
Wen et al. [58]	China	Mental health care	Single group ITS	Winning bids and volumes for SSRIs publicly set and mandatory	0	High	Volume, expenditure, and cost	SSRI volume increased by 77% while total expenditures fell slightly.
Wen et al. [57]	China	Hepatitis B patients	ITS	Public winning prices and volumes for entecavir and tenofovir	0	High	Volume, expenditure and cost	Drug volumes increased by 76%; expenditures fell by 45% and unit costs by 69%.
Sun et al. [46]	China	Pharmaceutical firms	DID panel data	Transparent tenders where only winners access public market	0	High	Profitability, costs and R&D	Bid winning firms experienced improved financial performance and lower promotion costs.
Jian et al. [62]	China	Cardiac patients	DID insurance claims	National stent procurement with reduced tender prices and mandated use	0	High	Treatment cost; stent use	Total AMI treatment costs for PCI with stent fell by 40%.
Fan et al. [51]	China	Orthopaedic patients	Retrospective cohort	NCP and NVBP for hip implants with public winning bids	0	High	Hospitalisation and implant cost	Total cost fell by 4000 US dollars after NVBP; implant cost decreased from 5264 to 1143 US dollars.
Li et al. [52]	China	Cardiac patients	Single hospital ITS	NVBP for knee implants with restricted hospital choice	0	High	Total hospitalisation expenses	Mean total expenses fell from 65,300 CNY to 34,500 CNY per case.
Jiang et al. [43]	China	Diabetic patients	ITS	Insulin specific NVBP with mandatory public sector adoption	0	High	Monthly volume and expenditure	Monthly volume rose while expenditure fell from 86.6 to 52.6 million CNY.
Yang et al. [59]	China	CVD patients	Affordability analysis	NVBP for statins with publicly negotiated generic prices	0	High	Unit prices; affordability	NVBP cut monthly costs from 0.24 to less than 0.02 days wage for targeted statins.
Chen et al. [49]	China	Hospital procurement	Pre post analysis	National tenders with pilot implementation in public facilities	0	High	Volume and expenditures	Winning product volume rose 16 fold; target drug spending fell by 37%.
Zhao and Wu [61]	China	Public hospitals	ITS	National NVBP pilot with binding results for hospitals	0	High	DDD cost and expenditure	Unit costs fell by 60% and expenditure by 63% for targeted drugs.
Huang et al. [41]	China	Chronic disease patients	Multilevel ITS	City wide implementation linked to insurance payment	0	High	Outpatient total expenditures	Outpatient expenditures fell by 16%–26% for hypertension and diabetes patients.
Li et al. [44]	China	Hospital procurement	Longitudinal study	National NCDP with mandated volume targets and rewards	0	High	Substitution to winning brands	Substitution towards bid winning brands occurred in 43% of drugs.
Wang et al. [48]	China	Hospital procurement	DID analysis	NCDP target vs. off list substitutable drugs	0	High	Sales value and volume	Selected drugs saw large price shifts; unselected substitutes captured market space, offsetting some savings.
Wang et al. [47]	China	Diabetic patients	ITS	National NVBP for oral antidiabetics with monthly tracking	0	High	Generic market share; prices	Overall prices fell by 60%; generic market share rose from 71% to 88%.
Wang et al. [55]	China	Clinical drug use	Single city ITS	Expanded NVBP results applied to public hospitals	0	High	Use of policy related drugs	NVBP sharply increased use of cheaper contracted products and reduced use of non winning brands.
Yang et al. [60]	China	Cardiac patients	ITS	City level stent procurement with binding winning prices	0	High	Stent prices and expenditure	Average prices of selected stents fell by more than 90%.
Lin et al. [54]	China	Hospital outpatients	DID analysis	NCDP for 54 antibiotics with hospital rewards	0	High	Antibiotic and injectable use	Limited impact on outpatient rates; inpatients showed substitution toward cheaper varieties.
Jian et al. [42]	China	CVD patients	Before after claims	National stent procurement with mandatory use	0	High	Expenditures; treatment mix	Total expenditure for PCI with stent cases fell by 23%.
Song et al. [45]	China	Diabetic patients	ITS	Combined NCDP and national price negotiation	0	High	Novel hypoglycaemic cost	Policy package produced sharp unit cost reductions for targeted novel agents.

## 5 Discussion

Passive interventions focused on sharing information do increase knowledge but rarely translate to service delivery improvement. This finding, which resolves much of the so‐called ‘transparency paradox’, is consistent with arguments that information without a credible accountability mechanism has limited influence on public systems [8, 65].

In contrast, VOICE and TEETH portfolios show that transparency can have sizeable yet conditional effects when it is tied to mechanisms that alter incentives. Intensive community monitoring in Uganda and Malawi and participatory budgeting with fiscal disclosure in Brazil are associated with higher utilisation, better quality and lower child mortality [11, 28, 33, 34]. Centralised drug and device purchasing in Colombia, Brazil, India and China similarly links transparent tenders to enforcement, and this combination coincides with lower prices and maintained or improved access [39, 40, 46, 50, 66].

At the same time, VOICE forums with little authority and TEETH schemes with weak enforcement or conflicting industrial goals often show flat outcomes, while Chinese procurement reforms reveal spillovers through substitution and non‐drug charges. These patterns highlight the role of contextual conditions C such as administrative capacity, regulatory coherence and provider autonomy in determining whether Voice and Teeth become operational or remain symbolic.

### 5.1 The Role of Contextual Conditions in Shaping Impact

The contextual variable (C) in our framework is not a passive backdrop but a decisive factor that determines whether transparency mechanisms become operational or remain purely symbolic. Our review identifies several specific examples where administrative and regulatory features either blunted or amplified the impact of reforms. In Tamil Nadu and Delhi, the success of TEETH interventions was rooted in strong institutional capacity, including the use of pre‐qualification procedures for suppliers, quality assurance inspections, and the relative autonomy of the central procurement agencies. These features ensured that sanctions for non‐compliance were credible and that the state possessed the technical ability to monitor large‐scale contracts.

In contrast, contextual conditions in other settings served to neutralise the potential gains of transparency. In Ethiopia and Tanzania, the impact of price disclosure was diminished by conflicting industrial policy goals that prioritised the growth of local manufacturers over patient affordability. In these cases, government purchasers sometimes paid higher prices for local products, which meant that transparency regarding lower import prices did not lead to procurement shifts. Similarly, the effectiveness of reference pricing in Tanzania was blunted by misaligned reimbursement ceilings and a lack of administrative capacity to audit private pharmacies. Within the VOICE portfolio, results from Brazil suggest that the presence of established participatory budgeting procedures provided the necessary institutional infrastructure for citizens to influence spending in ways that reduced infant mortality. These examples illustrate that for transparency to yield a payoff, the surrounding administrative and political environment must be capable of supporting the chosen accountability pathway.

### 5.2 Reframing Transparency in Health Financing Debates

The findings speak to three main debates in the literature on transparency and health governance.

First, they temper the expectation that additional disclosure will realign incentives on its own. Information appears necessary but rarely sufficient, a view that aligns with earlier conceptual work in public finance and social accountability [2, 3, 8]. Once interventions are coded by their VOICE and TEETH content rather than by their labels, the evidence base looks less contradictory than previous reviews sometimes suggested [4, 7].

Second, the review offers a more cautious reading of digital transparency in health finance. Post 2015 procurement portals, price tools and performance dashboards mostly operate as Passive Transparency, since they release data without clear institutional connections to citizen Voice or administrative Teeth. Their immediate effects on services appear modest unless they are explicitly wired into complaint handling, audit selection or participatory forums.

Third, the TEETH cases add nuance to discussions of fiscal openness as an anti corruption and expenditure management strategy. Transparency combined with credible enforcement can reduce rents and reorient spending toward cost effective options, but it can also change utilisation patterns and interact with industrial policy aims. This suggests that transparency instruments should be understood as components of broader institutional arrangements that balance affordability, access and quality rather than as simple devices for eliminating waste.

### 5.3 Implications for Policy and Practice

The empirical material has several implications for governments, development partners and practitioners who design transparency initiatives in health. A first implication concerns the limited direct effects of Passive Transparency. Portals, public notice boards and scorecards may still matter for reasons of normative commitment or future institutional development, yet the evidence does not support strong expectations that these tools alone will improve coverage, quality or equity. Where resources are constrained, investments that couple disclosure with stronger Mvoice or Mteeth appear more promising than expansion of stand alone information projects. A second implication comes from the VOICE portfolio. Interventions with the largest gains tend to combine facility specific information, repeated and facilitated deliberation, written follow‐up commitments and at least some influence over resources or management. In contrast, community forums that lack such levers usually leave utilisation and health outcomes unchanged. This suggests that ministries of health and donors should give more attention to institutional links between information, participation and decision making authority. A third implication concerns TEETH interventions. Centralised purchasing and volume linked reimbursement can rapidly lower drug and device prices, although success depends on credible contract management, monitoring capacity and willingness to sanction non‐compliance. Complementary measures such as clinical guidelines, safeguards against cost shifting and equity sensitive monitoring are needed so that fiscal savings do not come at the expense of stewardship or distributional goals.

### 5.4 Limitations

This review has several limitations. Our search was restricted to English‐language, indexed publications, so relevant evidence in other languages, local journals and grey literature may be under‐represented, with possible publication bias. The interventions and outcomes are highly heterogeneous, which precluded formal meta‐analysis and means that our synthesis is primarily interpretive rather than quantitative. Finally, classification of interventions into Passive Transparency, VOICE and TEETH involved some judgement, and any misclassification would likely blur differences between categories rather than create them.

### 5.5 Directions for Future Research

The findings of this review point to several priorities for future evaluation research. First, the evidence base remains uneven across regions and clinical areas. More rigorous studies are needed outside the current concentration in maternal and child health or pharmaceuticals, including work on underrepresented service sectors such as mental health services, chronic disease management, diagnostics and routine quality of care processes.

Second, a more direct test of the proposed conditional logic would come from designs that vary VOICE or TEETH intensity while holding the transparency input constant. Factorial designs, phased rollouts or comparisons of bundled versus unbundled interventions would help distinguish whether impact arises primarily from disclosure, from the accountability mechanism, or from their specific interaction.

Third, future studies should integrate routine administrative data, such as insurance claims, procurement transactions and facility registers, with credible counterfactual designs. This is especially relevant for TEETH reforms where unit price, volume and total expenditure may move in different directions and where spillovers can occur through substitution or non‐drug charges.

Finally, greater attention is needed to context as an empirical object rather than a residual category. Evaluations should document implementation capacity, such as the credibility of sanctions, the autonomy of procurement agencies and complaint handling functionality. Such research should also assess distributional effects to determine whether reforms improve equity or unintentionally shift financial burdens onto disadvantaged patients.

## 6 Conclusion

The review argues that gains from transparency in health service delivery are conditional rather than automatic. Once interventions are separated into Passive Transparency, VOICE and TEETH and contextual capacity is brought into the analysis, the heterogeneous findings on open government in health become more coherent. Information only reforms tend to improve awareness and change a limited set of processes but rarely transform what patients encounter in clinics or pharmacies. Larger and more durable gains appear where fiscal transparency is combined with empowered citizen Voice or credible state Teeth in settings where administrative and political conditions do not neutralise these mechanisms.

For policy actors and development partners, transparency is better viewed as a diagnostic input whose effects depend on the accountability arrangements surrounding it than as a cure for weak health systems. Investments in disclosure are likely to have greater impact when designed alongside institutional pathways that specify who can act on information, through which forums, with what authority and under which incentives. Recognising both the potential and the limits of transparency does not weaken the case for openness. Instead, it anchors that case in a realistic account of how incentives, institutions and everyday practices shape health service delivery.

For policy and practice, the central message of this review is straightforward: transparency is necessary but not sufficient for improving health service delivery. Information about budgets, procurement and performance only begins to matter when specific actors are able to act on it, through institutionalised channels of citizen Voice or state Teeth, and when contextual capacity does not neutralise those channels. Designing transparency reforms therefore requires moving beyond questions about which data to publish, towards questions about who can use that information, in which forums, with what authority and with what consequences for providers.

## Funding

The authors received no specific funding for this work.

## Ethics Statement

This study is a review of published literature and did not involve human participants or the use of identifiable personal data.

## Conflicts of Interest

The authors declare no conflicts of interest.

## Data Availability

Data sharing not applicable to this article as no data sets were generated or analysed during the current study.
